# Source dipole analysis reveals a new brain response to visual symmetry

**DOI:** 10.1038/s41598-020-79457-x

**Published:** 2021-01-11

**Authors:** John Tyson-Carr, Marco Bertamini, Giulia Rampone, Alexis Makin

**Affiliations:** Department of Psychological Sciences, University of Liverpool, Liverpool, L69 7ZA UK

**Keywords:** Extrastriate cortex, Attention

## Abstract

Visual regularity activates a network of brain regions in the extrastriate cortex. Previous EEG studies have found that this response scales parametrically with proportion of symmetry in symmetry + noise displays. The parametric symmetry response happens in many tasks, but it is enhanced during active regularity discrimination. However, the origins and time course of this selective enhancement are unclear. Here we answered remaining questions with new source dipole analysis. As assumed, the parametric symmetry response found at the sensor level was generated by a pair of dipoles in the left and right extrastriate cortex. This bilateral activity was itself enhanced during regularity discrimination. However, we identified a third, and later, symmetry response in the posterior cingulate during regularity discrimination. Unlike the extrastriate response, this previously unknown activation only indexes strong, task relevant regularity signals. This clarifies the neural circuits which mediate the perceptual and cognitive aspects of symmetry discrimination.

## Introduction

Visual symmetry, being prevalent throughout the plant and animal kingdom^1^, is a key factor in perceptual organization^2–4^, and symmetry perception is a rapid and efficient process^5–7^. Furthermore, visual symmetry can affect performance in secondary tasks^8,9^. This suggests symmetry itself is processed automatically, even when it is not task relevant. Beyond the role that symmetry plays in terms of perceptual organization, its evolutionary relevance is also key in that symmetry is more aesthetically pleasing and relevant for mate selection^10–16^, potentially due to perceptual fluency^17^.

The neural response to symmetry is restricted primarily to extrastriate regions of the visual cortex^18^, a finding that has been observed across many subsequent fMRI studies^19–23^. These fMRI studies all found a consistent symmetry response in the extrastriate cortex, most prominently in V4 and the Lateral Occipital Complex (LOC). Reflectional (mirror) symmetry is characterized by the mirroring of a pattern along an axis to produce regularity, although the replacement of components within the pattern can induce noise. The extrastriate symmetry response scales parametrically with the proportion of symmetry in the image (a variable termed PSYMM). Conversely, there is no parametric symmetry response in the primary visual cortex (V1) or V2. The extrastriate response is similar whether symmetry is task relevant or not, but it is sometimes enhanced during regularity discrimination tasks^24^.

The extrastriate symmetry response can also be measured with ERPs (for review see Bertamini et al.^25^). Norcia et al.^26^ isolated a response to symmetry in their steady state paradigm. This has since been measured with classic ERP techniques^27^ and this component is now commonly called the sustained posterior negativity (SPN). With the SPN, amplitude at posterior electrodes is more negative from around 220 ms when participants observe symmetrical compared to asymmetrical stimuli^28–31^. Like the extrastriate activation in fMRI studies, SPN amplitude also scales with PSYMM^32^. Makin et al.^24^ replicated SPN scaling across five tasks with different groups of participants. The parametric SPN was comparable when participants were discriminating regularity or some secondary stimulus dimension (such as element color, the pitch of a simultaneous sound, the orientation of a small overlaid triangle, or the spatial distribution of the elements, Fig. 13). However, the SPN was selectively enhanced in the Regularity task. Indeed, at 40% PSYMM, attention to regularity made the difference between presence and absence of an SPN.

Other labs have also addressed the automaticity of the SPN. Jacobsen and Hofel^27^ found that the SPN was only present when participants were performing an objective symmetry classification task but not an aesthetic evaluation task. However, recent reanalysis of this data by Jacobsen et al.^33^ suggests both stimulus and task driven effects in all tasks. Hofel and Jacobsen^29^ found the SPN was present when people were performing contemplation or oddball detection tasks. Other work has isolated the symmetry response in odd harmonics of the SSVEP^34^. This symmetry response is also observed during passive view conditions^35^. In summary, it is clear that the SPN (or related components) are generated by symmetry in a range of tasks, but can be selectively enhanced during active regularity perception. The same applies to the extrastriate symmetry response measured with fMRI^20^.

Although the selective enhancement in the Regularity task was robust in Makin et al.^24^, several questions remain. First, does the enhancement arise from increased activation of the extrastriate symmetry network itself, or does it arise from task-specific activation in other brain areas? Second, it is curious that a such large bilateral network responds to symmetry (e.g. V3a, V4 and LOC): perhaps some areas show more selective enhancement than others? Third, do such selective enhancements have distinct latencies? Source analysis in Makin et al.^24^ was preliminary, and the time courses of source-level activations were not examined. We thus exploited advanced source-dipole analysis to reanalyze the data from Makin et al.^24^ and answer these questions.

## Results

This investigation involved six analyses, which built on each other to reveal new properties of the brain response to symmetry. In Analysis 1, we re-examined the sensor-level ERP data from Makin et al.^24^. In addition to the classic bilateral SPN, we identified a new vertex positivity in the 80 and 100% PSYMM conditions of the Regularity task. In Analysis 2, we used dipole fitting to examine the potential cortical sources of these ERPs. As expected, the SPN was generated by left and right extrastriate dipoles. The new vertex positivity in the Regularity task was generated by a third dipole in the posterior cingulate cortex (PCC). In Analysis 3, we extracted source waveforms from these dipoles, and used permutation tests to identify intervals with a significant effect of PSYMM. In Analysis 4, we compared source waveforms across tasks, and found that the extrastriate response was itself significantly enhanced in the Regularity task. In Analysis 5, we used jackknifing procedures to establish the latency of onset and peak these source waves. This confirmed that the PCC response was a unique component, with its own distinct time course. Finally (and importantly), Analysis 6 replicated all results from the Regularity task of Makin et al.^24^ by reanalyzing data from a similar experiment by Palumbo et al.^32^.

### Analysis 1: sensor-level ERP analysis identifies a new vertex positivity component

**Figure 1:**
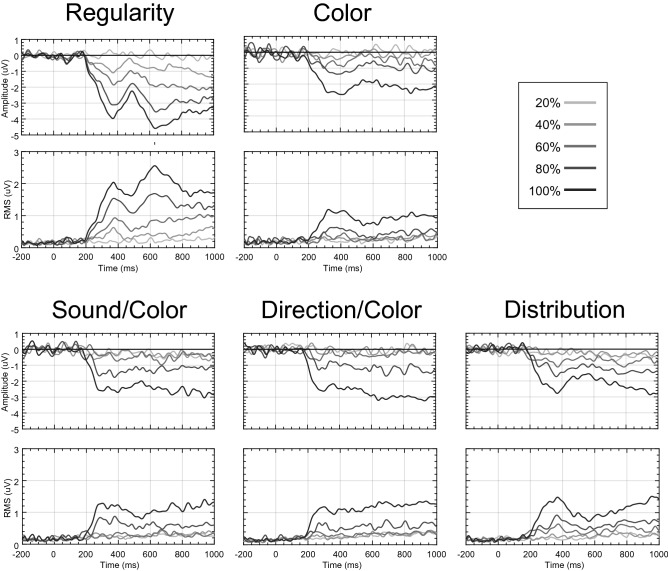
ERP and GFP for scalp level data. Top panels show grand average difference waves (PSYMM–random) at posterior electrode cluster [PO7, O1, O2 and PO8] for each task and level of PSYMM. The bottom panels show global field power (GFP) of the difference map.

As in Makin et al.^24^, we computed the difference between random trials (0% symmetry) and symmetry trials (20%, 40%, 60%, 80%, 100%). Figure 1 illustrates the SPN at posterior electrodes (top row) and global field power (GFP) for the topographic difference map for each task and level of PSYMM (bottom row). In all tasks, there was a strong effect of PSYMM on GFP after ~ 300 ms.

**Figure 2:**
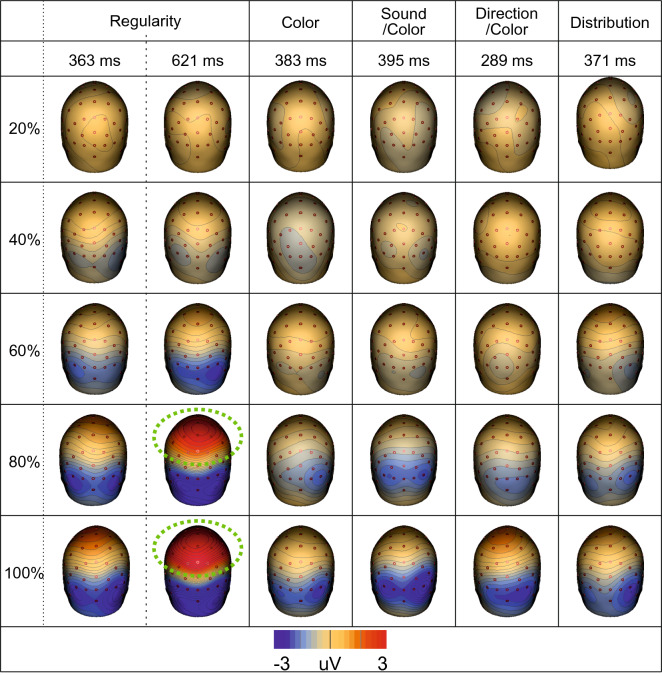
Scalp maps for each task and condition. Scalp maps at each distinct peak within the GFP for each task and each level of PSYMM. The vertex positivity is highlighted with green halos. Scalp maps were created using BESA 7.0 (https://www.besa.de/).

Figure 2 shows the topographic difference maps at each distinct GFP peak. The scaling of the SPN response with PSYMM is represented by increasing bilateral negativity (blue) across posterior electrodes. This effect was observed across all tasks, but it was enhanced in the Regularity task (for a more detailed sensor-level analysis, see Makin et al.^24^).

As can be seen in Figs. 1 and 2, the Regularity task was distinct in that it apparently elicited two separate GFP peaks. The first corresponds to the peak of the SPN at 363 ms. This scaled with PSYMM. However, the second peak in the Regularity task, occurring at 621 ms, is characterized by a strong positivity over the vertex of the scalp. This was only found in the 80 and 100% reflection conditions.

The topographic patterns at 363 ms and at 621 ms in the 80 and 100% conditions of the Regularity task were similar. Thus, the vertex positivity at 621 ms could simply be an extension of the cortical sources producing the SPN. Alternatively, a third cortical source could have generated the vertex positivity. Source dipole analysis was used to test these alternative possibilities, as described next.

### Analysis 2: dipole fitting reveals that the new vertex positivity is generated by a third source in the posterior cingulate cortex

**Figure 3:**
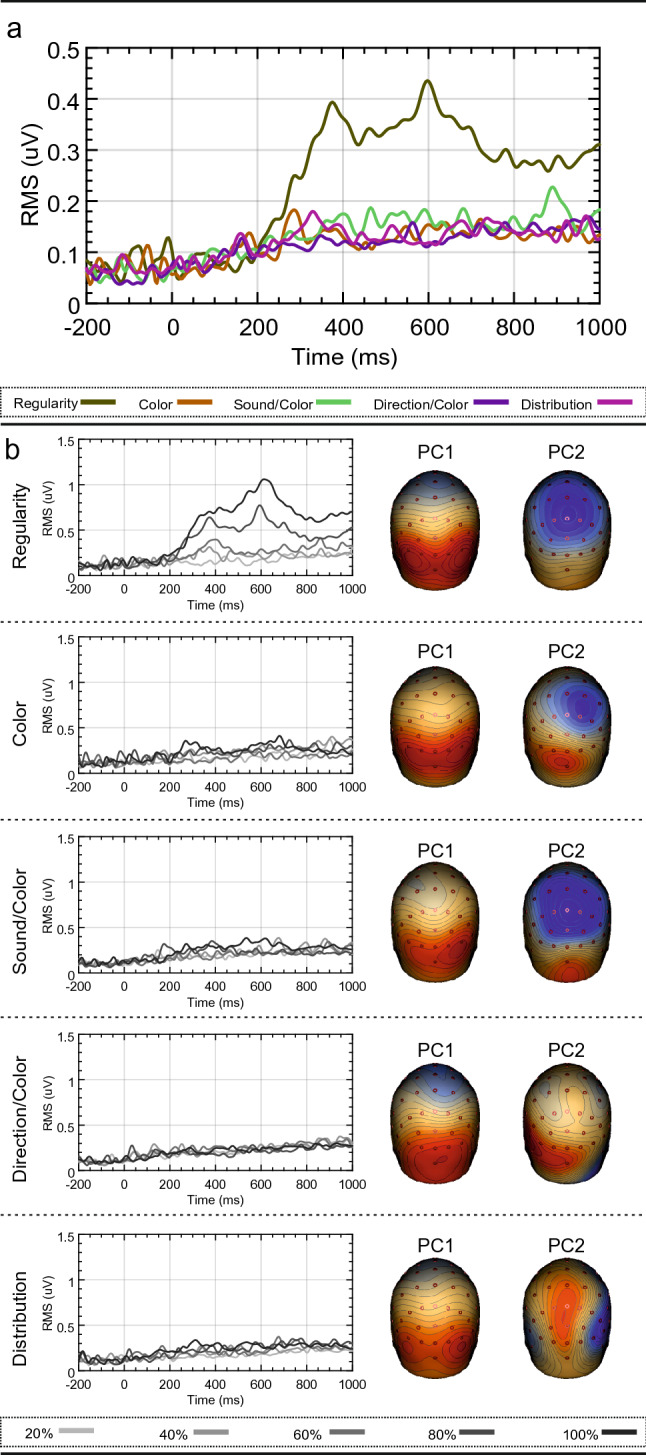
Residual variance for source dipole models comprising extrastriate sources only. (**a**) Residual variance GFP for the source dipole model for each task. (**b**) Residual variance GFP for each source dipole model and condition. Two principle components explaining the greatest amount of variance are also shown. Scalp maps were created using BESA 7.0 (https://www.besa.de/).

Analysis 1 identified a new vertex positivity in the 80 and 100% conditions of the Regularity task. In Analysis 2, we determined whether this was generated by a unique cortical source. Given prior knowledge of the cortical origins of the SPN, the first stage was to determine the extent to which the observed data could be explained by a source dipole model comprised of two bilateral ECDs in the extrastriate cortex. For each task, the residual variance not accounted for by the bilateral extrastriate source dipole model was calculated. Figure 3a shows the GFP of the residual variance for each task. In the Regularity task, there was a significant period of unexplained variance when only the two bilateral extrastriate ECDs were included. In contrast, there were no periods of unexplained variance in the other four tasks.

Furthermore, Fig. 3b illustrates the residual variance GFP for each condition. Only the 80 and 100% symmetry conditions of the Regularity task have unexplained variance. Importantly, the period of unexplained variance temporally coincides with the vertex positivity identified in Analysis 1.

PCA is a method employed in EEG data analysis to reduce the rank of multi-channel EEG data^36^, but it is useful for illustrating the brain components that explain the largest proportion of variance in the data. The two most significant principle components, explaining the largest proportion of variance, are shown as topoplots in Fig. 3b (note that the polarities of principle components are arbitrary and serve only to illustrate spatial distribution). The first principle component across all tasks was a scalp map typical of the SPN. However, the second principle component in the Regularity task was a scalp map with a maximum over the vertex. Thus, it appears that the positivity over the vertex in the Regularity task is its own discrete component with a unique cortical generator.

**Figure 4:**
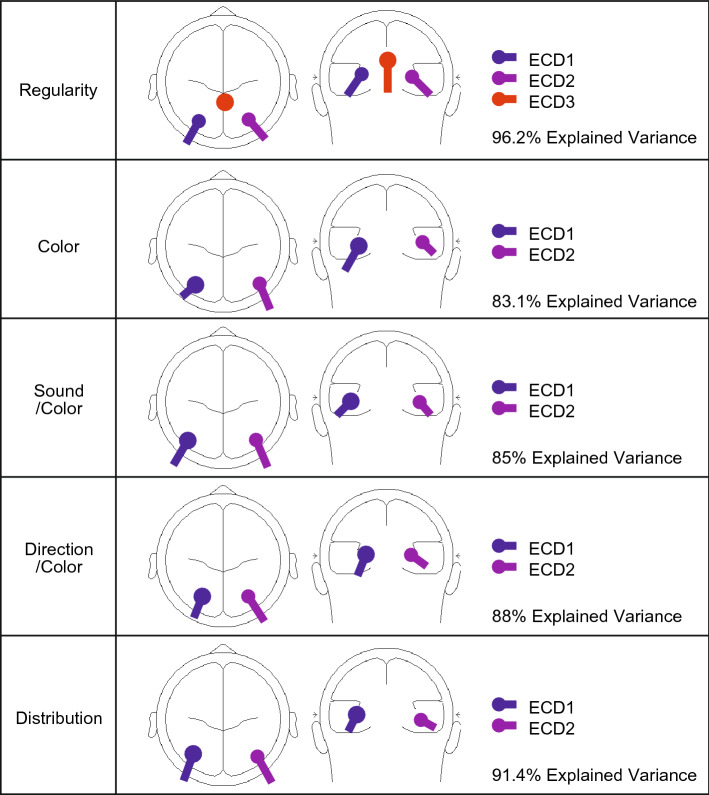
Glass brains for final source dipole models. Final source dipole models for each task and the corresponding amount of explained variance within the average difference waveform across all levels of PSYMM. Glass brains were created using BESA 7.0 (https://www.besa.de/).

Since a source dipole model comprising only extrastriate sources did not explain all observed scalp activity during the Regularity task, a third ECD was fitted. During the period of unexplained variance, activity appeared to originate in the vicinity of the posterior cingulate cortex (PCC). After including this third ECD in the PCC, the source dipole model explained 96.2% of variance (in contrast to only 87.3% when including only the two extrastriate ECDs). We thus finalized the five source dipole models, one for each task, that explained all significant portions of observed scalp activity. The final models are outlined in Fig. 4. All models included two ECDs placed bilaterally within the extrastriate cortices, with only the Regularity task requiring a third ECD placed within the PCC. After finalizing these source dipole models, the source waveforms for each participant, dipole and condition were exported to allow for statistical analysis.

**Figure 5:**
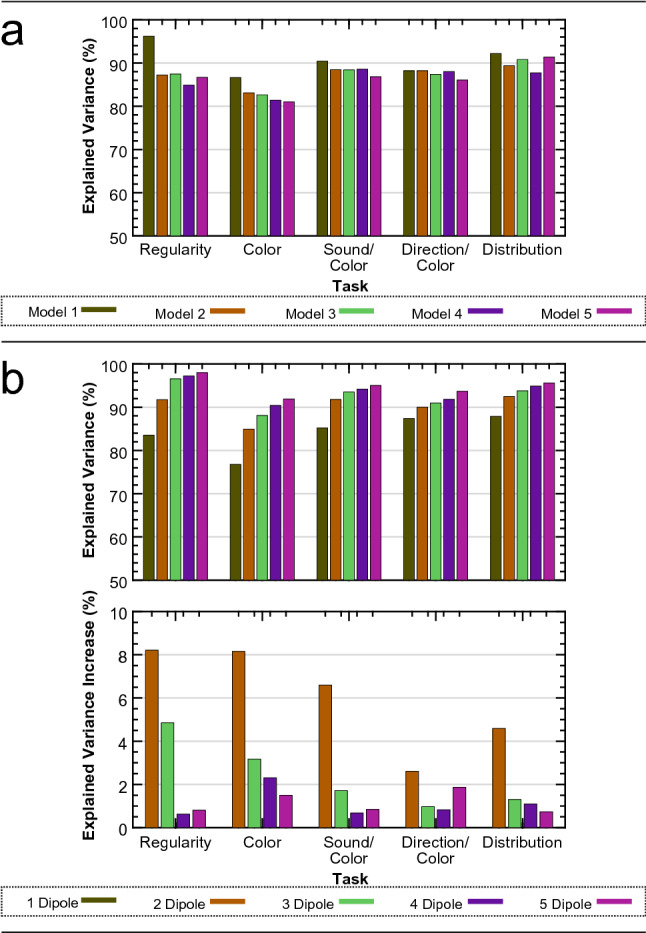
Source dipole model sufficiency testing. (**a**) Explained variance for the final five models fitted to the data from each task individually. (**b**) Explained variance for a new set of source dipole models comprising between 1 and 5 dipoles for each task. Total explained variance is shown (top) as well as an increase in explained variance from a dipole model comprising one less dipole (bottom).

Considering the suitability of the final model is important in source dipole modelling. To enable final model comparisons, we measured the fit of each model against the data from each task. The results are summarized in Fig. 5a. Firstly, the model obtained from the Regularity task increases the explained variance when fitted to data from all other tasks. This is most likely due to the presence of an extra dipole explaining a further proportion of the data. However, the increase in explained variance is minimal. Secondly, all models explain a similar proportion of variance when fitted to data from experiment 2, 3, 4 and 5, indicating that the individual models are very similar across experiments. A main visible difference is seen when the Regularity task data includes the third dipole, thus highlighting the importance of a third cortical source.

Although these results suggest that the third dipole is specific to the Regularity task, the Regularity task model was built specifically for the Regularity task data. This may account for the large increase in explained variance when fitted against the Regularity task data. Furthermore, as is visible from Fig. 3b, the second principle component for both the Color and the Sound/Color task is similar to the vertex activity we observed for the Regularity task, suggesting that the PCC source may be active during these tasks. To investigate this, we produced a set of new models with a varying number of dipoles for data from each task. Not only does this produce models specific to each dataset, it indicates what happens to the explained variance as more dipoles are included. Results are summarized in Fig. 5b. As expected, explained variance increases with the number of dipoles, but at a declining rate. Across all tasks, the inclusion of a second dipole is vital. This is obvious given the bilateral nature of the SPN. However, the inclusion of a third dipole has varying effects. Firstly, the third dipole has the largest influence on the data from the Regularity task, followed by the Color task and then the Sound/Color task. The large increase in explained variance from the third dipole in the Regularity task, as well as the minimal gain from a fourth and fifth dipole, emphasizes the sufficiency of a three-dipole model. With regards to the Color and Sound/Color tasks, the question arises as to whether the two-dipole model is the most appropriate, and, whether more dipoles should be included. For both tasks, including a third dipole increases explained variance by a small proportion. Furthermore, the increase is similar when we include a fourth or fifth dipole. Therefore, there is almost equal justification for a three-dipole model than there is for a four- or five-dipole model. Figure 3a or b highlight no significant period of unexplained variance with two-dipoles, suggesting that the most appropriate model would be a two-dipole model for the Color and Sound/Color tasks. The increasing explained variance from further dipoles is most likely due to the dipoles simply explaining noise within the data. If the second principle component from the Color and Sound/Color tasks does indeed represent the PCC activity, it may simply reflect momentary attention to the regularity within the images.

### Analysis 3: source waveform analysis reveals effects of PSYMM within each task

**Figure 6:**
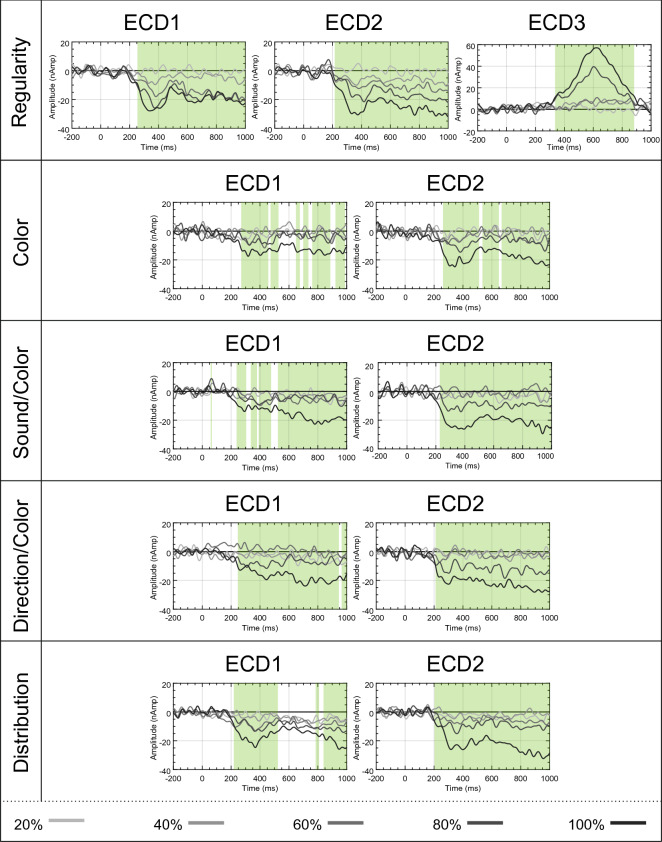
Source waveforms. Source waveforms for each ECD from each condition and task. Significant main effects of PSYMM are highlighted in green.

Analysis 2 identified bilateral ECDs in all tasks, and a unique PCC ECD in the Regularity task. The aim of Analysis 3 was to determine the sensitivity of these ECDs to PSYMM. Therefore, each ECD was submitted to a repeated-measures permutation-based ANOVA to identify intervals with significant main effects of PSYMM. In order to confine analysis to periods of substantial cortical activity, intervals demonstrating a significant effect were masked if the amplitude did not exceed a threshold. To define this threshold, the standard deviation across conditions was calculated for the pre-stimulus period^37^. The threshold was set to 5 times the standard deviation, so significant effects were masked if the signal was not 5 times greater than the reported noise. Figure 6 illustrates the source waveforms and the corresponding main effects of PSYMM highlighted in green for each ECD (P < 0.05).

**Figure 7:**
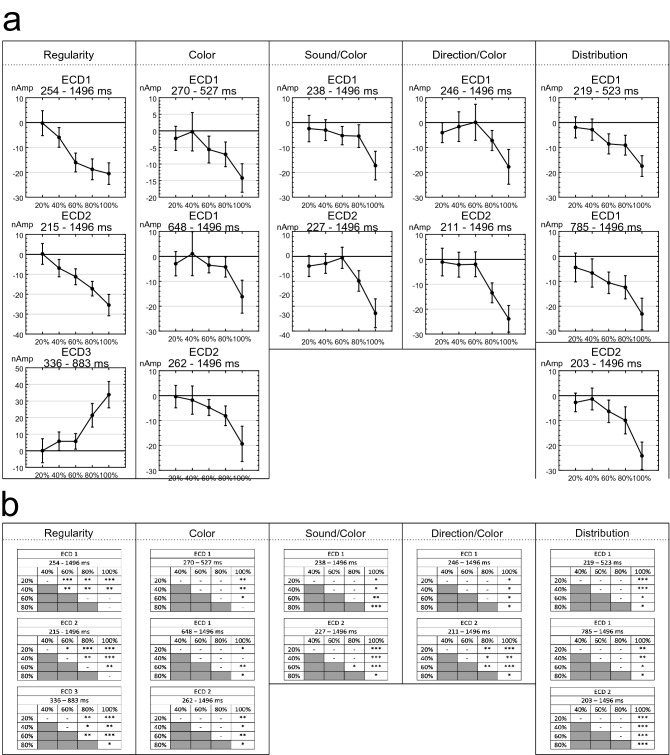
Mean activity in intervals and results of post-hoc testing. (**a**) Mean source activity averaged over latencies demonstrating significant main effects of PSYMM for each task and ECD. Error bars represent 95% within-subject confidence intervals. (**b**) Results of post-hoc permutation-based t test for each significant latency interval (*P < 0.05; **P < 0.01; ***P < 0.001).

To allow post-hoc comparison on PSYMM increments, source activity across distinct, continuous periods of significant differences identified by the permutation tests were extracted. The activity averaged in these intervals are displayed in Fig. 7a. A parametric response to PSYMM was observed in extrastriate ECDs. Conversely, ECD3 within the Regularity task was strongest in response to 80 and 100% symmetry.

Mean activity between all levels of PSYMM were compared using post-hoc permutation-based t tests. These utilized 5000 permutations and corrected for multiple comparisons using the tmax method^38^. Results from post-hoc tests are summarized in Fig. 7b. In the Regularity task, the parametric response to PSYMM in extrastriate sources is emphasized by increased sensitivity of the ECDs to changing levels of PSYMM. In contrast, significant differences in all other tasks are confined to comparisons with the 80 and 100% reflection condition.

### Analysis 4: source waveform comparison across tasks reveals that the Regularity task enhancement is present within extrastriate cortex

**Figure 8:**
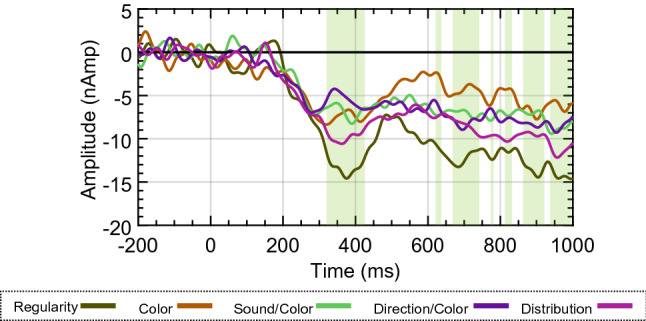
Average source waveforms across hemispheres. Mean source activity for each task averaged across both extrastriate sources. Significant main effects of Task are highlighted in green.

Makin et al.^24^ reported an enhanced SPN response during the Regularity task at the sensor level. However, the contribution of the third PCC source to scalp activity during this task may explain the sensor level enhancement. To investigate this further, activity across the left and right extrastriate sources were averaged. Firstly, the extrastriate activity across the 300–1000 ms interval was averaged and submitted to a mixed-effects ANOVA with Task and PSYMM as factors. A significant main effect of Task on activity was found [F(4,125) = 2.638, *P* = 0.037, η_p_^2^ = 0.078], and also of PSYMM [F(2.858,357.275) = 77.036, *P* < 0.001, η_p_^2^ = 0.381]. Next, the averaged extrastriate activity was submitted to a permutation mixed ANOVA with Task and PSYMM as factors. Figure 8 shows clusters of main effects of Task (*P* < 0.05). Figure 8 shows enhanced activity for the Regularity task between 320 and 426 ms, and also sporadic effects throughout the remainder of the epoch.

In summary, Analysis 3 shows that activity within the extrastriate cortices was enhanced when attending to regularity, and the SPN enhancement observed in Makin et al.^24^ is not simply due to summated activity from the third PCC source at the sensor level.

### Analysis 5: time course of the 3 cortical responses to symmetry

An advantage of EEG is the excellent temporal resolution. This allowed us to estimate the timing of the observed neural responses. We utilized the jackknifing method for estimation of component timing^39^.

The onset of cortical activity for each ECD was defined using a percent-amplitude latency measure, i.e. the point at which the amplitude exceeds a certain percentage of the component’s peak amplitude. As recommended by Liesefeld^40^, the threshold was set to 30% of the peak amplitude and the onset/offset of cortical activity was determined based on this threshold for each ECD.

**Figure 9:**
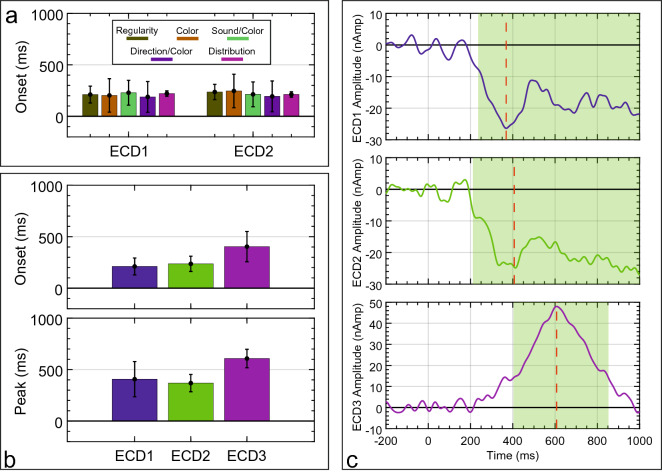
Mean latencies for source waveforms. (**a**) Mean onset for ECD1 (left extrastriate) and ECD2 (right extrastriate) across all tasks. (**b**) Mean onset and peak latency for each ECD in the Regularity task. (**c**) Average source waveform across all participants for each ECD in the Regularity task. Component time-course using a 30% amplitude threshold is highlighted in green and peak latency is indicated by a red dotted line.

First, the mean onset of extrastriate sources (ECD1 and ECD2) was extracted for each task and submitted to a mixed ANOVA. Figure 9a illustrates the mean onset for each extrastriate source and task. No significant main effect of Task on onset latency was observed [F(1,125) = 0.08, *P* = 0.778, η_p_^2^ < 0.001], and there was no other main effect or interaction (*P* > 0.981). This suggests that Task requirements had no effect on the latency of the extrastriate symmetry response.

Second, to compare the time-course of the extrastriate sources with the PCC source observed in the Regularity task, the onset and peak latency for ECD1, ECD2 and ECD3 were analyzed (see Fig. 9b,c). There was a borderline effect of ECD on onset latency [F(1.085,27.123) = 4.105, *P* = 0.05, η_p_^2^ = 0.141]. There was also a significant main effect of ECD on peak latency [F(1.018,25.439) = 4.726, *P* = 0.039, η_p_^2^ = 0.159]. Due to the sustained nature of the extrastriate source activity, there was no clear offset for ECD1 or ECD2. In contrast, ECD3 in the PCC had a mean offset at 825.764 ms (± 106.151 ms). This analysis reveals that PCC source is a distinct component, with its own unique time-course.

### Analysis 6: regularity task effects replicated with reanalysis of Palumbo et al.^32^

**Figure 10:**
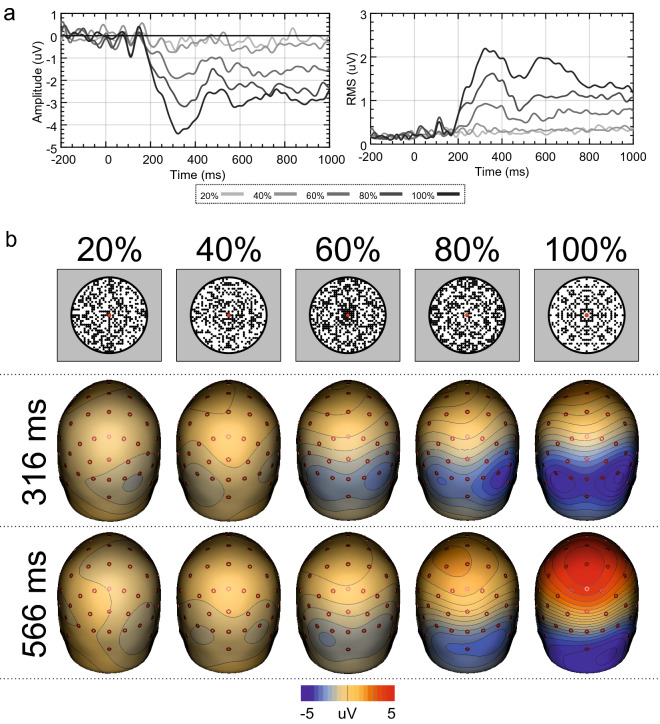
ERP, GFP, scalp maps and stimuli from Palumbo et al.^32^. (**a**) Mean activity at electrodes PO7, PO8, O1 and O2 and GFP across all electrodes for the different levels of PSYMM obtained from a reanalysis of Palumbo et al.^32^. (**b**) Scalp maps at the peak activity within the GFP, alongside the stimuli used in this experiment. Scalp maps were created using BESA 7.0 (https://www.besa.de/).

Makin et al.^24^ used five separate tasks with varying demands. However, the Regularity task, which produced both an enhanced extrastriate response and a third PCC ECD, was also used in a previous study^32^. Palumbo et al.^32^ also used the same PSYMM levels, but with different stimuli (Fig. 10). Therefore, we reanalyzed the data from Palumbo et al.^32^ to determine whether similar cortical activation patterns were present. Analysis 6 found that all important effects from the Regularity task of Makin et al.^24^ were replicated with reanalysis of Palumbo et al.^32^.

As reported by Palumbo et al.^32^, the SPN response increased in magnitude with the level of symmetry (see Fig. 10). The GFP again showed two peaks, in this data set (Fig. 10a).

**Figure 11:**
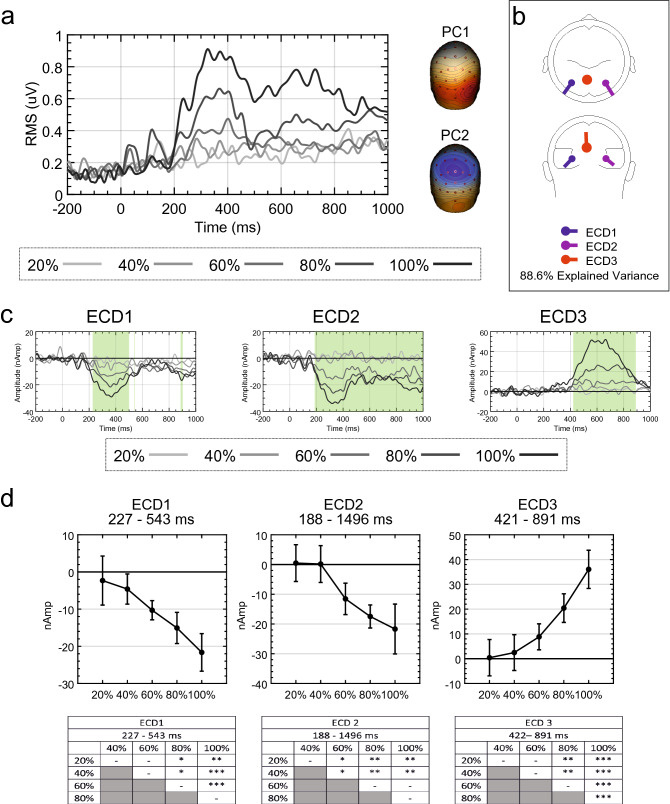
Source dipole model residual variance, source waveforms and mean activity in intervals for Palumbo et al.^32^. (**a**) Residual variance GFP for each condition when including only extrastriate sources and the two principle components explaining the greatest amount of variance. (**b**) Final source dipole model and proportion of explained variance. (**c**) Source waveforms for each ECD from each condition. Significant main effects of PSYMM are highlighted in green. (**d**) Mean source activity averaged over latencies demonstrating significant main effects of PSYMM for each task and ECD. Results of post-hoc testing are also shown (*P < 0.05; **P < 0.01; ***P < 0.001). Scalp maps and glass brains were created using BESA 7.0 (https://www.besa.de/).

As can be seen from Fig. 11a, a source dipole model comprising bilateral extrastriate sources leaves a large proportion of residual variance, but only within the 80 and 100% conditions. Furthermore, the two principle components explaining the most variance were identified. Again, PC2 represents a cortical activation over the vertex (Fig. 11a). After fitting a third ECD, which was again localized to the PCC, explained variance increased from 79.9 to 88.6% (Fig. 11b).

The resulting source waveforms for each ECD and condition are illustrated in Fig. 11c. The results of the permutation tests are highlighted in green (*P* < 0.05). In the left extrastriate cortex source (ECD1), the effect of PSYMM arises at approximately 227 ms following stimulus onset and dissipating at 543 ms. In the right extrastriate cortex source (ECD2), the PSYMM effect begins at 188 ms and continues through to the end of the epoch at 1000 ms. In the PCC source (ECD3), the effect of PSYMM is observed between 422 and 891 ms. Mean source activity between the most substantial periods of significant differences for each ECD was averaged, and this mean activity is displayed in Fig. 11d. The results of post-hoc tests are also displayed. These results are similar to those of the Regularity task in Makin et al.^24^, described in Analysis 3.

**Figure 12:**
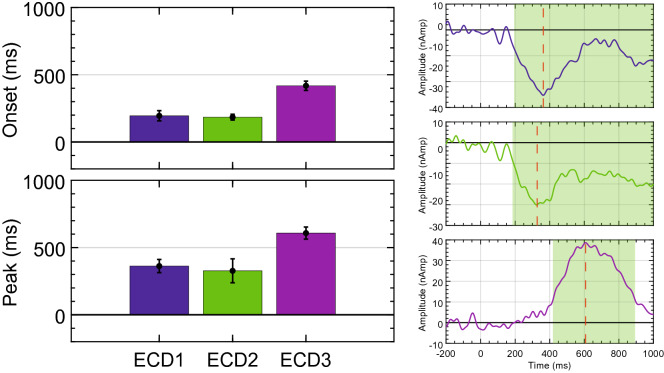
Mean latencies of source waveforms for Palumbo et al.^32^. Mean onset and peak latency for each ECD from Palumbo et al.^32^. Average source waveforms across all participants for each ECD are also shown. Component time-course using a 30% amplitude threshold is highlighted in green and peak latency is indicated by a red dotted line.

Using the jackknifing procedure, component onset/offset were again extracted using a 30% amplitude threshold. Mean onset and peak latencies are shown in Fig. 12 for each ECD. Similar to the Regularity task of Makin et al.^24^, a main effect of ECD on onset latency was observed [F(1.506,34.635) = 72.261, *P* < 0.001, η_p_^2^ = 0.759]. Furthermore, a significant main effect of ECD on peak latency was observed [F(1.081,24.861) = 24.208, *P* < 0.001, η_p_^2^ = 0.513]. No discernible offset was present for extrastriate sources; however, the PCC source had a mean offset at 893.555 ms (± 92.727 ms). This analysis again closely parallels the results from the Regularity task of Makin et al.^24^, and confirms that the PCC source is a distinct, and previously unknown, response to symmetry.

## Discussion

The SPN wave measured at posterior electrodes scales with PSYMM in several tasks, but is selectively enhanced during regularity discrimination^24^. However, the cortical origins and time course of this selective enhancement were previously unclear. Here we gained new insights with source dipole analysis. We found that the SPN was generated by two dipoles in the left and right posterior extrastriate cortex, as expected. Activity at these bilateral dipoles scaled with PSYMM in all tasks, but was enhanced in the Regularity task. This suggests the SPN enhancement in the Regularity task was caused by increased activation within the extrastriate symmetry network. Source analysis did not suggest any further fractionation of the extrastriate response, which was always captured by two bilateral dipoles. There were no additional extrastriate dipoles generated in the Regularity task explaining the selective enhancement. Although this could reflect poor spatial resolution, Keefe et al.^20^ also observed attentional enhancement across the whole network with fMRI.

There was, however, a third dipole during the 80 and 100% conditions of the Regularity task. This dipole was located outside the visual cortex in the PCC. The PCC response was distinct from the extrastriate response. Firstly, the new PCC dipole was generated later in the trial, after the extrastriate enhancement was already evident. Secondly, the PCC dipole was not sustained, and instead had a clear offset. Finally, it was only found in the 80 and 100% PSYMM conditions of the Regularity task, rather than scaling smoothly with PSYMM. This PCC was also found in the data from Palumbo, et al.^32^, so we can be confident that it was not an anomaly recorded in one data set.

Previous fMRI measurements of the symmetry response have used visual cortex regions of interest, so they would not capture this PCC activation^18,20,21,23,41^. Sasaki et al.^22^ did run a whole-brain analysis which revealed little symmetry-related activity outside of the visual cortex. However, this was carried out on data from their passive viewing task, and we found no PCC dipole under such conditions.

An important consideration from previous EEG research is whether activity in the PCC can be reliably measured with EEG methods, due to the distance between the surface of the scalp and the PCC^42^. However, using EEG source localization methods, previous research has identified oscillatory activity from the PCC which correlated with BOLD responses within other regions of the default mode network^43^. Although this was investigated with respect to the default mode network, it highlights the ability to measure activity from the PCC reliably with EEG. Similarly, time–frequency data measured from other portions of the cingulate gyrus have been shown to correlate with BOLD response in the same regions using simultaneous EEG and fMRI methods^44,45^. Despite the limited spatial resolution of EEG, we proceed with the assumption that the third dipole was in the PCC. What is its functional significance? Previous work suggests that the PCC is a highly connected communication hub, possibly tuning attention and shifting between broad and narrow attentional states^42^. A role for the PCC in detecting motivationally significant environmental changes has been suggested^46^. Mohanty et al.^47^ found a correlation between PCC activity and the speed of attentional shifts to motivationally significant stimuli. The authors also revealed increased connectivity between the PCC and intraparietal sulcus, another important region in spatial attention^48^. Finally, Soon et al.^49^ reported that PCC voxel patterns could be used to predict whether people would voluntarily respond with the left or right button. Given this previous work, the PCC activation in our Regularity task is explicable: the regularity task required participants to integrate attended visual information before choosing which button to press.

Makin et al.^24^ and Palumbo et al.^32^ speculated that perceptual decisions about symmetry involve applying a binary threshold to the continuous extrastriate response. If the symmetry response crosses the threshold, participants would report some regularity. Conversely, if the symmetry response falls below threshold, participants would report no regularity. It is likely that the bilateral extrastriate sources index the continuous symmetry response. Perhaps the PCC is involved in making binary decisions? This is plausible, but we note that the PCC activation was only evident in cases where (a) there was a clear symmetry signal and (b) participants were engaged in a task where this signal mattered (i.e. in 80 and 100% conditions of the Regularity task). Participants were also making decisions about the presence/absence of symmetry on other trials during the Regularity task. Therefore, the PCC activation may capture just one cognitive aspect of perceptual decision making—namely, registration of a strong symmetry presence when such a signal is behaviorally relevant.

There are occasions where symmetry is behaviorally relevant in real world settings. To give one example, Tyler^50^ speculated that in natural ancestral environments reflectional symmetry was a reliable visual clue that another symmetrical organism, such as a potential predator or conspecific, has us in its line of sight. The PCC may be involved in communicating this important symmetry signal to the rest of the brain, enabling us to react appropriately.

A common theme in consciousness research is that unconscious neural processing remains local, but conscious representations are broadcast around a global thalamocortical network^51^. Given this, it is possible that all symmetry representations are processed locally within the extrastriate cortex, but when symmetry becomes task relevant, there is connectivity with the non-visual PCC as the information is broadcast to other brain regions.

It is likely that there is bidirectional flow of information between the bilateral extrastriate cortex and PCC. However, the *cognitive* PCC may not be responsible for top down attentional enhancement of the *perceptual* extrastriate response. After all, the extrastriate response was already enhanced *before* the PCC was activated. Furthermore, the extrastriate enhancement happened in 40 and 60% PSYMM conditions, where there was no PCC activation. The extrastriate enhancement thus seems independent from the PCC activation.

The neural architecture supporting perceptual decisions regarding symmetry were also investigated by Kohler et al.^41^. Using fMRI-informed EEG source imaging, the authors revealed an overlapping set of regions that were involved in both encoding and decision-related processes for symmetry perception. Not only did areas V3v, V4, VO1 and VO2 exhibit encoding related, stimulus-locked responses to rotational symmetry, they also exhibited significant decision related, response-locked activity. These findings emphasize the importance of considering decision-related activity that can be parsed from encoding-related activity. We suggest the PCC mediates an aspect of decision related activity which has not been identified in previous neuroimaging work.

Finally, we note that the vertex positivity resembles the well-studied P3b component: many studies have found that infrequent oddball targets generate a positive wave at central-parietal electrodes from 300 to 800 ms. It could be that 80 and 100% symmetry served as infrequent oddballs in the Regularity task. Perhaps the vertex positivity is closely related to the P3b? We think this is unlikely for three reasons. First, the P3b is not generated by a single dipole in the PCC^52^. Second, the P3b peaks earlier in most oddball experiments. Finally, in additional (preliminary and unpublished) analyses we have also looked at other SPN data sets and found that PCC dipole can be generated by symmetries that are not infrequent oddballs. In summary, we do not think the PCC response can be reduced to the P3b.

Following from Makin et al.^24^, we conclude that the extrastriate network is the cortical generator of the SPN. We conclude that this response is automatic: it is generated regardless of whether symmetry is the attended feature or not. However, the extrastriate response can be enhanced by attention. We have also discovered a new response to symmetry in the non-visual posterior cingulate cortex. The posterior cingulate could index strong symmetry signals when they are task relevant, and communicate this to other parts of the brain.

## Methods

As originally described in Makin et al.^24^, 130 participants were recorded across five different tasks (26 participants in each). The study and all procedures were approved by the Research Ethics Committee of the University of Liverpool. All procedures followed were in accordance with both institutional and national guidelines for ethical standards on human experimentation, as well as the Helsinki Declaration as revised in 2013. All participants gave written informed consent. EEG was recorded using the BioSemi Active-2 system with 64 electrodes arranged in accordance with the internationally recognized 10–20 system.

**Figure 13:**
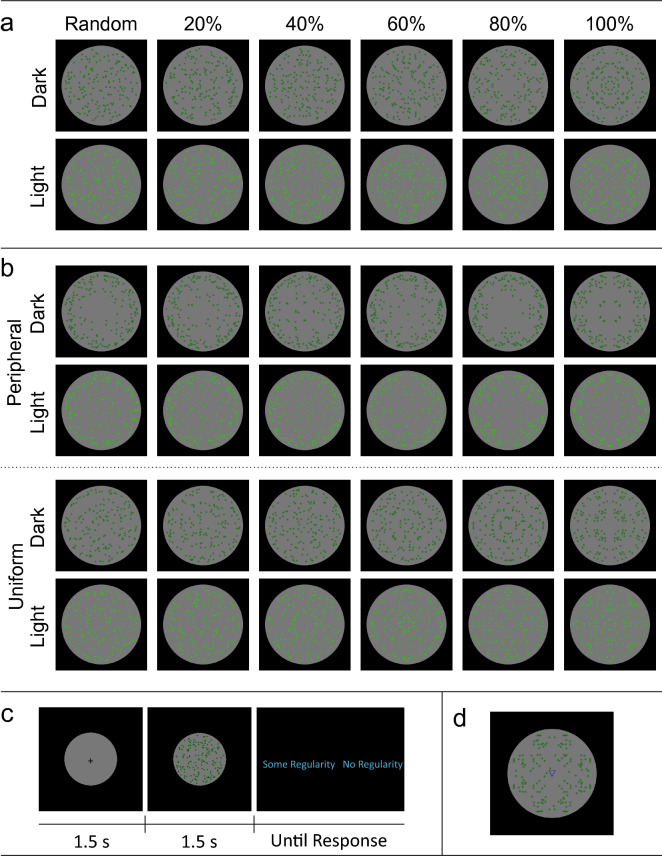
Stimuli and procedure from Makin et al.^24^. (**a**) Patterns used in the regularity, color, sound/color and direction/color tasks. Columns illustrate increasing PSYMM, rows indicate dark/light green stimuli. (**b**) Stimuli used in distribution task. (**c**) Experimental paradigm for regularity task. All other tasks differed only in the binary judgement required (light green/dark green; congruent/incongruent; uniform/outside). (**d**) Stimulus with directional triangle from direction/color task.

Stimuli from Makin et al.^24^ are shown in Fig. 13. These varied from 0% (random) to 100% (perfect symmetry) in increments of 20%. For the 600 images used in each task, 300 were random, and 60 belonged to each level of PSYMM (20%, 40%, 60%, 80%, 100%). Stimuli were presented in a different, randomized sequence for each participant. The Regularity and Color tasks used one stimulus set, the Sound/Color and Direction/Color tasks used a second stimulus set, and the Distribution task implemented a third stimulus set.

On each trial, a 1.5 s fixation period was followed by a 1.5 s pattern presentation. Following the pattern, a binary judgement was required. In the *Regularity task*, the choice was between “some regularity” and “no regularity”. In the *Color task*, a choice was between “light” and “dark” (these options referred to the shade of green). In the *Sound/Color task*, a low pitch (200 Hz) or a high pitch (800 Hz) beep accompanied the pattern presentation. The choice was between “congruent” and “incongruent” (congruent trials were those where light green dots were paired with high pitch beeps or dark green dots were paired with low pitch beeps: incongruent trials were those where light green dots were paired with low pitch beeps or dark green dots were paired with high pitch beeps). In the *Direction/Color task*, an upward or downward pointing triangle was presented at fixation. Again, the choice was between “congruent” and “incongruent” (congruent trials were those with light green dots and an upward facing triangle or dark green dots and a downward facing triangle: incongruent trials had light green dots with a downward facing triangle or dark green dots with an upward facing triangle). Finally, in the *Distribution task*, the choice was between “uniform” or “outside”. Uniform trials were those with uniformly distributed dots, whereas outside trials presented patterns with dots disproportionately distributed around the periphery (for detailed methods and behavioral results, see Makin et al.^24^).

### ERP analysis

Before extracting the source dipoles, the ERP data from Makin et al.^24^ was reanalyzed with an improved pipeline. EEG data were pre-processed using EEGLAB 14.1.2b^53^ in MATLAB 2019a (MathWorks Inc., USA). Data were band-pass filtered from 0.1 to 25 Hz, downsampled to 256 Hz, and re-referenced to the scalp average. Independent component analysis (ICA) was used for artefact correction. Independent components representing eye-blinks and other artefactual data were removed.

Event-related potentials for each condition were computed by averaging − 500 to 1496 ms epochs with − 199 to 0 ms pre-stimulus baseline. Trials were excluded if amplitude at any electrode within the epoch interval exceeded ± 100 μV. The mean number of trials submitted for analysis per participant was 586 ± 11.6 in the Regularity task, 576 ± 23.4 in the Color task, 572 ± 21.5 in the Sound/Color task, 551 ± 50.4 in the Direction/Color task and 569 ± 42.7 in the Distribution task.

The SPN at each level of PSYMM was defined as the difference between the corresponding PSYMM waveform (20%, 40%, 60%, 80%, 100%) and the random waveform. This provided a difference wave representing the neural response to symmetry with varying levels of noise. All ERP data is available on Open Science Framework (https://osf.io/qp3sm/).

### Source dipole reconstruction

In order to investigate the spatiotemporal dynamics of the brain response to symmetry, a source dipole model was constructed in BESA v. 7.0 (MEGIS GmbH, Munich, Germany). For the greatest accuracy, it is necessary to utilize data with a large signal-to-noise ratio. Thus, a grand average potential was computed by averaging the difference (symmetry–random) waveform across all levels of PSYMM for the purpose of defining the model. This was done for each task separately, producing five separate grand average waveforms.

Producing an appropriate source dipole model involved fitting equivalent current dipoles (ECDs) to describe the three-dimensional source currents in the brain regions contributing predominantly to the data. To identify an appropriate number of ECDs to fit, principal component analysis (PCA) was first used to give a general indication of the number of sources contributing to the data. Given prior knowledge that the SPN is generated in the extrastriate cortex^22,23^, the first step was to fit bilateral ECDs within the extrastriate area. If a significant proportion of variance remained unaccounted for by the extrastriate ECDs, or if the PCA indicated other significant contributing sources, further ECDs were fitted using a sequential strategy^54,55^.

Classical LORETA analysis recursively applied (CLARA), which is an iterative application of the LORETA algorithm^56^, was then used to confirm and adjust the locations of the ECDs in the final model. Due to differences in gyral anatomy and functional representation in the cortex, ECD orientation can vary extensively between participants. Furthermore, ECD orientation has a larger impact on scalp activation patterns than ECD location, and a single ECD can model activity from several cubic centimeters of cortex^57^. Therefore, orientations of the ECDs were fitted with the constraint of fixed dipole locations and determined over a time interval spanning the source’s activation for each participant individually. Although the models were built using the grand average waveform averaged over all levels of PSYMM for the greatest signal-to-noise ratio, ECD orientation was determined using the 100% reflection condition across all latencies since this produced the strongest symmetry response. A 4-shell ellipsoid head volume conductor model was employed using the following conductivities (S/m = Siemens per meter): Brain = 0.33 S/m; Scalp = 0.33 S/m; Bone = 0.0042 S/m, Cerebrospinal Fluid = 1 S/m.

Source waveforms for each level of PSYMM and for each task were exported and analyzed using permutation-based repeated-measures ANOVA in the EEGLAB toolbox^53^. This used 5000 permutations.

Finally, latency estimates for the source waveforms were calculated using a jackknife-based method with a 30% amplitude threshold^39^. To implement this procedure, the peak amplitude in the latency interval 300–1000 ms was extracted. Next, the time point at which the waveform first reached 30% of this amplitude was described as the onset of the component, whilst the time point at which the waveform returned to this threshold was described as the offset. In line with jackknife methods, this was done on the waveform averaged across subjects to increase signal-to-noise ratio. Although using the averaged waveform across subjects precludes the estimation of component timing for individual participants, it is possible to extract individual subject estimates. Jackknifing implements a leave-one-out procedure whereby latencies are extracted for each of *n* waveforms comprising data from all but one of the subjects’ waveforms, where *n* is the number of subjects. Since each of these waveforms include data from *n-1* subjects, the latency is much less influenced by noise. Furthermore, the variance between the waveforms provides a vague estimation of the individual subject timings. These individual estimates would usually require specially designed statistical tests to allow statistical analysis^40^. However, we implemented the protocol outlined by Smulders^58^ which implements a simple transformation on the subaverage latencies and produces estimates that can be analyzed with typical statistical tests. The jackknifing protocol and retrieval of individual estimates was carried out using the toolbox described in Liesefeld^40^. Source waveforms and latency estimations are available on Open Science Framework (https://osf.io/qp3sm/).

## Data Availability

Code for all aspects of data analysis, as well as raw data, are available on Open Science Framework (RRID: SCR_017419) at https://osf.io/qp3sm/.
